# Bimetallic Nanowires on Laser-Patterned PEN as Promising Biomaterials

**DOI:** 10.3390/nano11092285

**Published:** 2021-09-02

**Authors:** Jana Pryjmaková, Markéta Kaimlová, Barbora Vokatá, Tomáš Hubáček, Petr Slepička, Václav Švorčík, Jakub Siegel

**Affiliations:** 1 Department of Solid State Engineering, University of Chemistry and Technology Prague, 166 28 Prague, Czech Republic; pryjmakj@vscht.cz (J.P.); polivkoa@vscht.cz (M.K.); slepickp@vscht.cz (P.S.); svorcikv@vscht.cz (V.Š.); 2 Department of Microbiology, University of Chemistry and Technology Prague, 166 28 Prague, Czech Republic; vokataa@vscht.cz; 3 Biology Centre of the Czech Academy of Sciences, SoWa National Research Infrastructure, Na Sádkách 7, 370 05 České Budejovice, Czech Republic; hubacektom@gmail.com

**Keywords:** surface modification, nanostructure, polymer, bimetallic nanowires, biocompatibility, antibacterial properties

## Abstract

As inflammation frequently occurs after the implantation of a medical device, biocompatible, antibacterial materials must be used. Polymer–metal nanocomposites are promising materials. Here we prepared enhanced polyethylene naphthalate (PEN) using surface modification techniques and investigated its suitability for biomedical applications. The PEN was modified by a KrF laser forming periodic ripple patterns with specific surface characteristics. Next, Au/Ag nanowires were deposited onto the patterned PEN using vacuum evaporation. Atomic force microscopy confirmed that the surface morphology of the modified PEN changed accordingly with the incidence angle of the laser beam. Energy-dispersive X-ray spectroscopy showed that the distribution of the selected metals was dependent on the evaporation technique. Our bimetallic nanowires appear to be promising antibacterial agents due to the presence of antibacterial noble metals. The antibacterial effect of the prepared Au/Ag nanowires against *E. coli* and *S. epidermidis* was demonstrated using 24 h incubation with a drop plate test. Moreover, a WST-1 cytotoxicity test that was performed to determine the toxicity of the nanowires showed that the materials could be considered non-toxic. Collectively, these results suggest that prepared Au/Ag nanostructures are effective, biocompatible surface coatings for use in medical devices.

## 1. Introduction

Advances in manufacturing and processing technologies have resulted in polymers being used in a wide range of medical and pharmaceutical applications [1,2]. These applications include artificial blood vessels, orthopaedic implants, surgical instruments, wound dressing and drug delivery. As most of these uses involve contact with the body, the polymers must have highly-tailored surface properties. Together with surface chemistry, properties such as surface morphology, roughness and wettability have a crucial influence on the interaction between an implant and a human body. These properties also play a significant role in ensuring that a medical device functions correctly and that its introduction does not lead to inflammation [3] or bacterial infection [4,5]. In particular, bacteriosis can occur due to many reasons ranging from the simple insertion of foreign material to the inadequate sterilisation or poor storage of a medical device.

Various surface modification techniques can be used to prevent bacteriosis. These include drug impregnation [6], the addition of an antibacterial agent during polymerisation [7] or the application of anti-adhesive/antibacterial coatings [8,9,10]. Generally, organic compounds are used as antibacterial coatings. Although nanometals are gaining attention for use in antibacterial coatings with an organic base, the use of pure metal coatings remains rare.

In this context, laser surface texturing (LST) is an excellent technique for the preparation of a platform suitable for the anchoring of metallic nanostructures. Under specific conditions, LST can create a laser-induced periodic surface structure (LIPSS), or so-called ripples, on the polymer surface [11]. The most important conditions are that the laser emits ultraviolet (UV) light and that the polymer substrate absorbs in the UV range (e.g., presence of structures with aromatic compounds) [12]. Thus, ripple patterns can be created on the surfaces of various polymers such as polyether ether ketone (PEEK) [13], polystyrene (PS) [14], polyethylene terephthalate (PET) [15] and polyethylene naphthalate (PEN) [16]. The ensuing ripple structure not only provides higher surface roughness and wettability but also changes the surface chemistry in ways that are important for implant–tissue interaction. 

Moreover, those structures offer the possibility of creating metallic nanowires using a vacuum evaporation technique (VET). VET is an effective physical vapour deposition technique for metallic nanomaterial synthesis. This technique offers several advantages including the control of layer thickness and a wide choice of substrates. Thus, while the applicability of a pure polymer is restricted in some ways, the use of a polymer–metal nanocomposite can extend the range of potential medical and pharmaceutical applications. Furthermore, metallic nanostructures can introduce novel physical and biological properties [1]. The use of certain metallic agents enables the preparation of biocompatible materials that can support cell proliferation [17,18,19] and antibacterial activity [9,20,21]. Metals such as copper [22], silver [23] and gold [24] are well known for their efficient antibacterial activity. However, metallic nanostructures can also be toxic in nanoparticle [25,26] or nanowire [27] form. When they exert an unfavourable influence on tissue, they can inhibit the proliferation of healthy cells and even kill them.

In this work, we focus on the surface modification of PEN in such a way that the resulting material is both biocompatible and antibacterial. To achieve this, we combined silver as the antibacterial agent and gold as the biocompatible material. A 248 nm KrF excimer laser and vacuum evaporation were used to prepare the Au/Ag nanowires. Using a combination of cytotoxicity and drop plate tests, we show that our material is biocompatible and demonstrates antibacterial effects against both Gram-positive (G^+^) and Gram-negative (G^−^) bacteria.

## 2. Materials and Methods

### 2.1. Materials and Apparatus

In this study, a foil of polyethylene naphthalate (PEN, thickness of 50 μm, supplied by Goodfellow, Ltd., Huntington, UK) was used as a substrate. The samples, having 1 × 1 cm^2^ areas, were cleansed of impurities using a stream of nitrogen and then modified using the linearly polarised light of a KrF laser (COMPexPro 50 F, Coherent, Inc., Santa Clara, CA, USA). Polarised light with a 248 nm wavelength was created using a UV-grade fused silica prism (model PBSO 248-100). The ripples were irradiated under the following conditions: 6000 pulses, pulse duration 20–40 ns, frequency 10 Hz and an aperture with an area of 5 × 10 mm^2^. The incidence angle of the laser beam was set to 0°, 22.5° and 45°, and laser fluency was changed to 4.5, 5.9 and 7 mJ·cm^−2^ with respect to the increased angles as well as the resulting changes in the irradiated areas.

After the modification, the Au/Ag nanowires (Au/AgNWs) were deposited onto the patterned PEN using vacuum evaporation apparatus LEYBOLD-Heraeus (Univex 450, Cologne, Germany). Evaporation was performed at room temperature at a pressure of 3 × 10^−4^ Pa, a deposition rate of 0.33 nm∙s^−1^ and a glancing angle of φ = 70° with respect to the sample surface, using resistively heated tungsten crucible. The deposition was carried out in two steps: Firstly, the gold was deposited onto one side of the ripples, the samples were rotated 180° and then the silver was deposited on the other side of the ripples. Both metals were supplied by Safina, a.s., Vestec, Czech Republic in the form of pellets (3.18 × 3.18 mm^2^, purity 99.99%). The thickness of the deposited metals (20 nm of gold corresponded to Δf = 490 Hz; 20 nm of Ag corresponded to Δf = 640 Hz) was monitored in situ by the oscillation of the quartz crystal. For thickness analysis, the scratch method was used [28]. A silica glass substrate was deposited simultaneously with the polymer samples. Next, scratches were created on the glass surface in five different positions and measured by atomic force microscopy (AFM VEECO CP II, Veeco Instruments, Inc., New York, NY, USA). Thickness variations did not exceed 5%.

### 2.2. Analytical Methods

The surface morphology of PEN, modified PEN (PEN 0/22.5/45°) and modified PEN with Au/AgNWs (Au/Ag PEN 0/22.5/45°) was analysed by atomic force microscopy (AFM VEECO CP II, Veeco Instruments, Inc., New York, NY, USA). The AFM images were taken using a Digital Instruments CP II setup. Samples were tacked to the sample holder using double-sided adhesive tape. An oxide-sharpened, P-doped RTESPA-CP silica probe (spring constant of 0.9 N·m^−1^, Veeco Instruments, Inc., New York, NY, USA) was used at an approximate resonant frequency of 300 Hz. The probe was attached to a flexible micro-cantilever. The ‘taping mode’ was chosen for measurement to minimise the damage of the sample surfaces. To obtain the representative data, four areas were scanned with a line scanning rate of 0.5 Hz. The same AFM method was used to measure parameters including surface roughness (*R_a_*), periodicity (*Λ*) and height (*h*). The surface roughness *R_a_*, characterised by the mean roughness value, represents an arithmetic average of the deviation from the centre plane of the sample. The periodicity (a repetition of ripples/nanowires) was measured as the distance between the two nearest bases/tops of these nanostructures. The heights of ripples/nanowires were measured from the bases to the tops. The parameters as *R*_a_, *Λ* and *h* were evaluated in ten different positions, then arithmetical means and standard deviations were calculated. Characteristic values of roughness (average roughness *R*_a_), as well as periodicity and height, were obtained from AFM scans in a software NanoScope Analysis v1.8. Variations of these parameters did not exceed 5%.

Samples with deposited Au/AgNWs were also visualised using scanning electron microscopy (SEM). The metal–polymer interface was detected by a focused ion beam (FIB), model SEM LYRA3 GMU (FIB-SEM, Tescan, Brno, Czech Republic). FIB cuts were made by a Ga ion beam. Investigated samples were polished using a lower beam current to provide a clean and flat surface. Images were taken at an angle of 54.8° with a voltage of 5 kV.

The concentrations of gold (Au) (3d), silver (Ag) (3d), carbon (C) (1s) and oxygen (O) (1s) on pristine, modified and metal-coated sample surfaces were determined using angle-resolved X-ray photoelectron spectroscopy (AR-XPS). For these measurements, an ESCAProbeP spectrometer (Omicron Nanotechnology GmbH, Taunusstein, Germany) was used. A monochrome X-ray beam with an energy of 1486.7 eV was used as a source. The photoelectron spectra were measured stepwise with a step of 0.05 eV. The samples were analysed at angles of 90° (perpendicular to the sample) and 14° from the left (+) and right (-) sides with respect to the sample surface. The spectra were evaluated by CasaXPS software. The concentration of elements was given in %. 

Energy-dispersed X-ray spectroscopy with a 2 mm 2SDD (SEM-EDS, X-MaxN, Oxford Instruments, Abingdon-on-Thames, UK) detector was used to clarify the Au-Ag interface. Before measurement, samples were coated with a gold layer with a thickness of 20 nm and secured with conductive carbon tape to discharge them. For the analyses of elements, an electron beam with a voltage of 10 kV was used. Data were evaluated using AZtecEnergy software v3.1. 

As the PEN was coated with metals possessing optical activity, absorption in the range of 350–900 nm was studied using the UV-Vis spectrometer model Lambda 25 (Perkin-Elmer, Inc., Waltham, MA, USA). Due to the presence of periodic arrays, absorption spectra were measured using polarised light with a perpendicular orientation to the nanowires. To obtain the polarised light, a WP25L-UB polariser (250–4000 nm) supplied by Thorlabs was added in front of a beam output. The scanning rate was set at 240 nm∙min^−1^. Data were evaluated using Perkin-Elmer UV WinLab software v4.2. 

To observe changes in wettability, contact angles were measured using goniometer model KRÜSS DSA 100 (KRÜSS, Hamburg, Germany) using a sessile drop method. An automatic pipette applied 10 drops of 2 µL of distilled water to the surface of the PEN (used in a pristine form), the PEN 0/22.5/45° and the Au/Ag PEN 0/22.5/45°. Subsequently, the contact angle was calculated using KRÜSS Advance software v2.0 using a three-point method. An arithmetical mean and standard deviation were determined from obtained values. 

Inductively coupled plasma mass spectroscopy (ICP-MS) was used to study the impacts of Ag^+^ and Au^n+^ ions on the antibacterial effect of the final material. To determine the concentrations of the released ions, leachates were created under the same conditions as in the antibacterial tests (see below). Unmodified PEN and modified PEN with Au/AgNWs were immersed into 5 mL of physiological solution (PS, 0.9% NaCl) at 24 °C for 3 and 24 h. Control leachates were prepared in the same way. All liquid samples were prepared in triplicate. Before analysis, each leachate was centrifuged using an Optima MAX-XP ultracentrifuge (Beckman Coulter, Brea, CA, USA) at an overload of 200,000 g for 30 min to eliminate the eventual residua of solid metals. The concentrations of Ag^+^ and Au^n+^ ions were measured using an Agilent 8800 triple-quadrupole spectrometer (Agilent Technologies, Santa Clara, CA, USA) with an auto-sampler. A MicroMist device with a peristaltic pump was used to homogeneously inject the sample into the atomiser.

### 2.3. Antibacterial Tests

The study of the antibacterial activity of the prepared samples was performed using a drop plate method of counting viable bacteria [29]. Antibacterial activity was observed against two bacterial strains: Gram-negative (G^−^) *Escherichia coli* (DBM 3138) and Gram-positive (G^+^) *Staphylococcus epidermidis* (DBM 3179). Bacteria were cultivated at 37 °C in an orbital shaker overnight. Prepared inocula were serially diluted in sterile physiological solution (PS), and optical density was measured at 600 nm (OD_600_). Next, samples were immersed into 1 mL of PS and inoculated with 1.1 × 10^4^ colony-forming units (CFUs) per 1 mL of *E. coli* and 2.2 × 10^4^ CFUs per 1 mL of *S. epidermidis*. Simultaneously, control samples of *E. coli* and *S. epidermidis* in PS without polymer samples were prepared. All samples were prepared in triplicate and incubated under static conditions (24 °C, test tubes standing on the laboratory table) for 3 and 24 h. Next, aliquots of 25 μL from each sample were intensively mixed by Vortex, and 5 drops were instilled onto thirds of the pre-dried agar plates. Agar LB (Luria-Bertani) for *E. coli* and Agar PCA (plate count broth) for *S. epidermidis* were used as cultivate media. After overnight cultivation on agar plates, the numbers of CFUs of both bacteria strains were counted, and mean values with standard deviation were calculated. The whole experiment was carried out under sterile conditions.

### 2.4. Cytotoxicity

The toxicity of PEN 0/22.5/45° with Au/AgNWs was tested on human primary lung fibroblasts. Human primary lung fibroblasts (MRC-5) were obtained from the American Tissue Culture Collection (ATCC, Manassas, VA, USA) and cultured in Minimal Essential Medium supplemented with 2 mM L-Glutamine (a stable dipeptide, Sigma-Aldrich, St. Louis, MO, USA) and 10% foetal bovine serum (FBS, Thermo Fisher Scientific, Waltham, MA, USA). The cells were maintained at the exponential phase of growth under specific conditions (37 °C, 5% CO_2_ and 95% humidity).

For the cell viability study, PEN 0/22.5/45° and Au/Ag PEN 0/22.5/45° were chosen. The viability of MRC-5 cells was determined using the WST-1 test [30], which is based on the transformation of tetrazolium salt (WST-1 reagent) into coloured formazan. Cell culture plates (6-well, VWR, Radnor, PA, USA) with inserted samples were sterilised with 70% ethanol of 99.9% purity for 30 min. Subsequently, samples were rinsed with sterile phosphate buffer (PBS, pH 7.4) and inoculated with 30,000 cells per cm^2^ in 2.5 mL of the complete cultivating medium in triplicate. Samples were cultivated for 24, 48 and 72 h. Next, the medium was removed, cells were rinsed with PBS, and WST-1 reagent diluted with complete phenol red-free DMEM was added. After cells were incubated for 2 h, the medium for cell cultivation was transferred onto 96-well plates (100 μL per well, 4 wells per sample), and absorbance was measured. Measurement was accomplished at 450 nm and the reference value was measured at 650 nm. Cells cultivated on standard tissue culture polystyrene (TCPS) were used as a control.

## 3. Results and Discussion

### 3.1. Surface Characterisation

**Figure 1:**
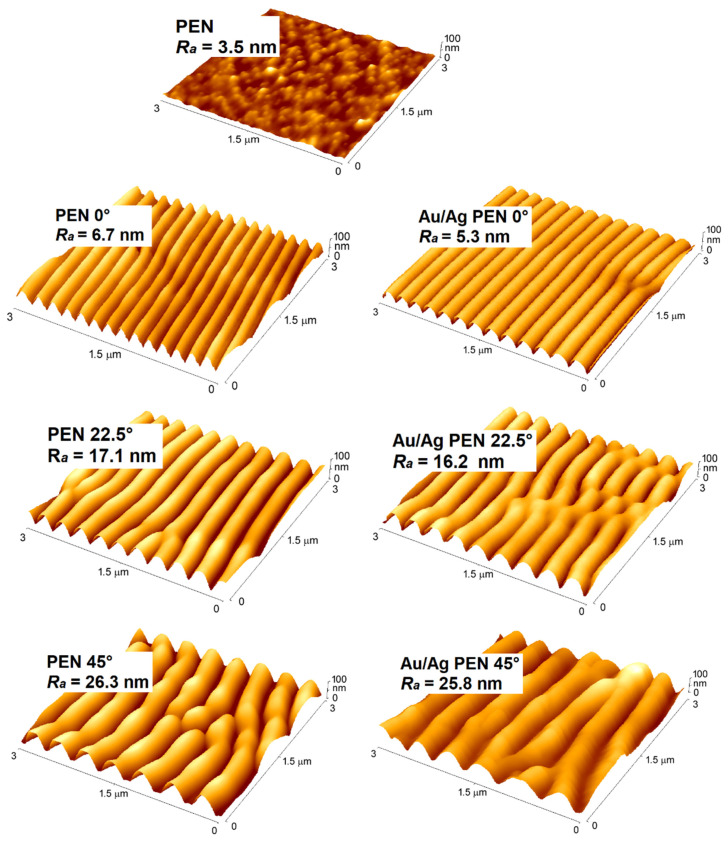
AFM 3D scans of PEN, PEN 0/22.5/45° and Au/Ag PEN 0/22.5/45° samples with corresponding values of surface roughness (*R_a_*).

Surface morphology was studied on pristine PEN (PEN), PEN modified under different incidence angles of laser light (PEN 0/22.5/45°) and modified PEN coated with bimetallic nanowires (Au/Ag PEN 0/22.5/45°). Laser irradiation with KrF lasers resulted in the development of periodical arrays on the PEN, with structural parameters differing at specific light incidence angles of 0°, 22.5° and 45° (see Figure 1). The incidence angle of the laser beam had a crucial influence on the surface roughness, periodicity and height of the final nanostructures, which are summarised in Table 1. It is obvious that increasing the incidence angle promoted surface roughness. This phenomenon has been also observed in the studies of Slepička et al. [31,32], where different values of roughness were obtained by changing laser fluency, and angle-dependent nanopatterning was created. The values for structure periodicity and height showed the same trend, which is in agreement with Equation (1) [33]:

(1) $$\Lambda =\frac{\lambda }{n-\sin\theta }$$

where λ represents the wavelength of the laser light used, n is the effective refractive index of the environment and θ is the incidence angle of the laser beam. The nanopatterning occurs due to the interference of the polarised incident beam and the scattered wave beam, which cause inhomogeneous intensity distribution on the surface and leads to a stable thermal process in the form of LIPSS. Generally, the interaction of the laser beam with the polymer surface is a complex mechanism involving a number of processes [34].

In particular, the Au/Ag PEN 0/22.5/45° samples provided interesting results; their roughness was slightly lower compared to modified PEN without bimetallic nanowires. This is due to a well-described phenomenon known as the shadow effect [35]. The evaporated material, at an angle of 70° with regard to the crucibles, condensed preferentially on one side of the periodical arrays and thus filled in free space between ripples. Therefore, in the case of bimetallic nanowires where gold and silver were deposited on both sides, the difference in surface roughness was less pronounced compared to monometallic AgNWs [16].

**Figure 2:**
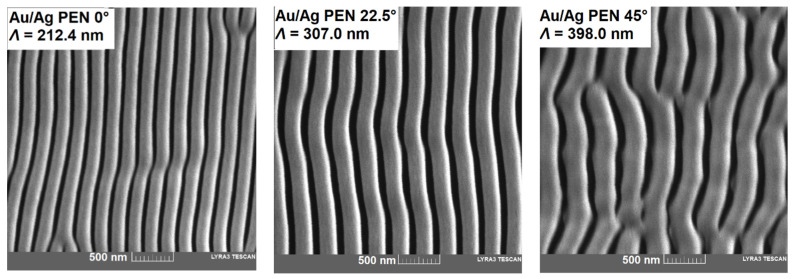
Micrographs from SE microscopy of Au/Ag PEN (0/22.5/45°) samples with corresponding values of periodicity (*Λ*).

**Figure 3:**
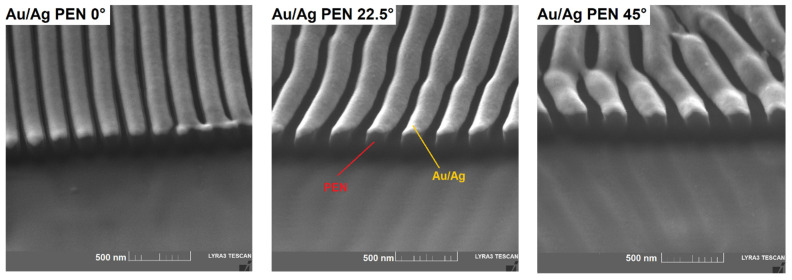
Micrographs from FIB-SEM showing metal–polymer interfaces on the surfaces of Au/Ag PEN (0/22.5/45°) samples.

To obtain more detailed information on the sample morphology, SE micrographs and FIB cuts were made on the surfaces of modified and metal-coated samples. Results from SEM confirmed the presence of periodically arranged nanostructures (Figure 2). The trend of increased periodicity once the incident angle was higher is obvious, which is in correlation with the periodicity derived from AFM. However, the localisation of metals on the PEN surface was not clear from the SE micrographs. Therefore, FIB-SEM analysis was performed to reveal the metal-polymer interface (see Figure 3). Due to two-step deposition, the homogeneous distribution of metals was clearly noticeable. Nevertheless, FIB-SEM analysis did not provide information about the respective distribution of gold and silver. For this reason, AR-XPS and EDS spectroscopies were carried out to determine the surface elemental composition.

**Figure 4:**
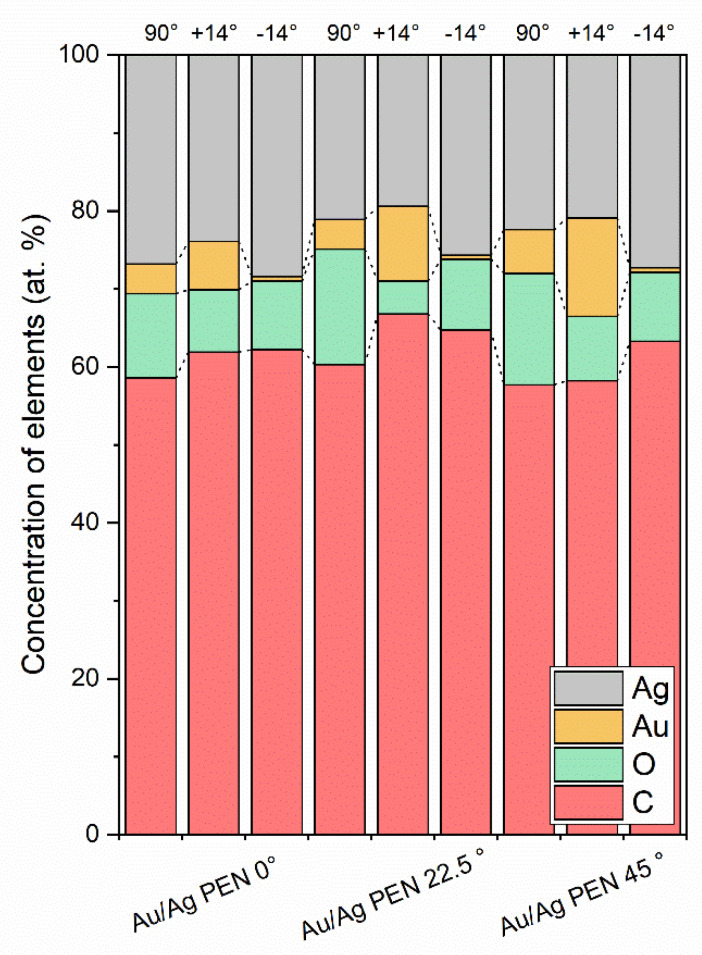
The concentrations of Au, Ag, C and O on Au/Ag PEN 0/22.5/45° samples determined by AR-XPS.

Results from the AR-XPS analysis at angles of 90° and ±14° (with respect to the sample surface) are summarised in Figure 4. While at 90°, analytical information could be determined from about 8–10 nm, detection at 14° provided information regarding chemical composition from a very small surface area (1–2 nm) [36]. AR-XPS showed inhomogeneous distribution of metals, which is consistent with the presence of bimetallic nanowires. Slightly higher silver concentrations on the surface of the Au/Ag PEN were due to the fact that gold was deposited on the modified PEN first, followed by silver in the second step. Thus, gold was covered by silver on the ripple tops, which led to higher concentrations of silver, especially at the angle of −14°.

**Figure 5:**
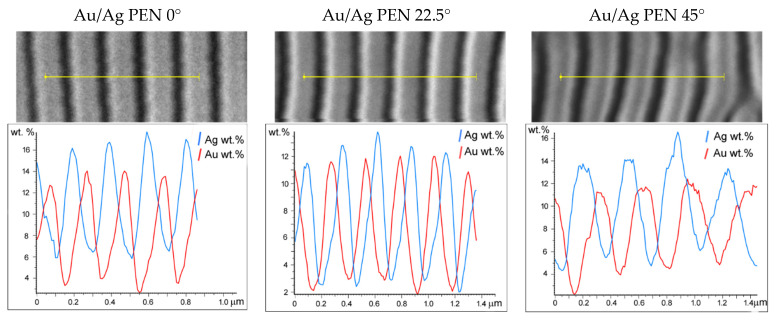
The concentration profiles of Ag and Au obtained by EDS spectroscopy in addition to SE micrographs of Au/Ag PEN 0/22.5/45° samples of corresponding areas.

For further understanding of the metal nanowire distribution, samples were observed using EDS spectroscopy. The distribution of individual metals is visible in the concentration profile shown in Figure 5. One can see that due to the two-step process, gold was deposited directly onto the modified polymers, while silver slightly covered Au nanowires, especially on the tops of the periodic structures. For this reason, the ripple tops showed higher concentrations of silver. Nonetheless, detected concentrations of gold and silver were similar within individual samples, pointing to the homogeneous distribution of metals in bimetallic nanowires.

**Figure 6:**
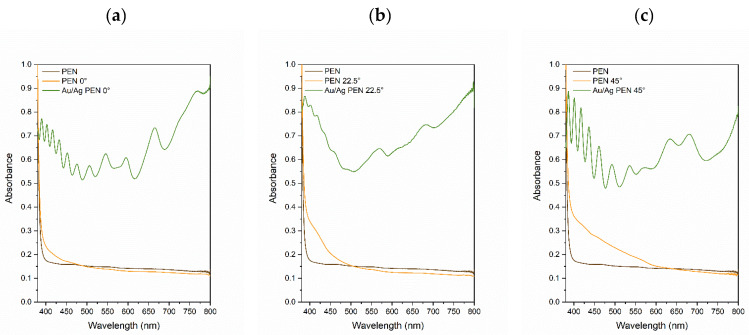
UV-Vis spectra of PEN, modified PEN and PEN with Au/AgNWs at incidence angles of (**a**) 0°, (**b**) 22.5° and (**c**) 45°.

As polymer samples were deposited by optically active metals, their absorbance was characterised using UV-Vis spectroscopy (see Figure 6). Nanostructured metals are known for surface plasmon resonance (SPR), which is affected by the oscillation of conduction band electrons [37]. This phenomenon is accompanied by the development of the SPR absorption band in UV-Vis spectra. Most metals have their SPR maxima in the range of 10–400 nm (e.g., silver); however, the SPR peaks of some metals (e.g., copper, gold) are found in the range of 400–800. In the case of composited materials and alloys, the SPR peaks are combined, which leads to the phenomenon known as coupling. Some similarity with our results (Figure 6) can be found in the study of Gunwidjaja et al. [38], where the authors observed the plasmon coupling of silver and gold peaks on AgNWs samples coated with an AuNPs/polyallylamine composite. Compared to Au/Ag PEN 0° and Au/Ag PEN 22.5°, the red-shift was evident on Au/Ag PEN 45° (Figure 6c) where the maximum SPR band was in the range of 650–700 nm. Except for plasmon coupling, pronounced interference at higher energies (350–500 nm) was observed in the case of all three samples (Au/Ag PEN 0/22.5/45°). This interference can be used to determine the layer thickness using the procedures given in [39].

**Figure 7:**
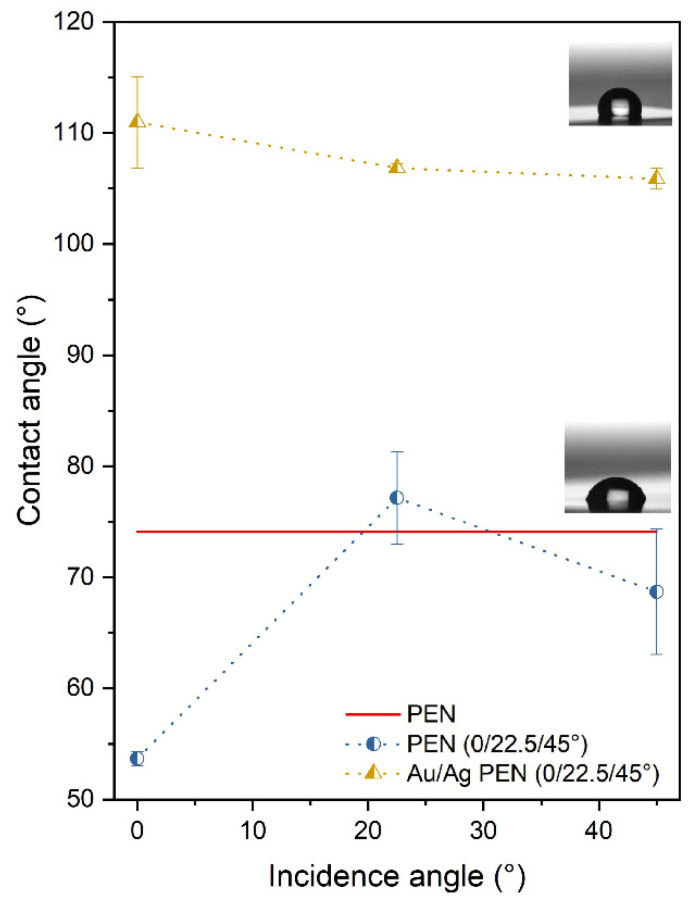
Contact angles of PEN, PEN 0/22.5/45° and Au/Ag PEN 0/22.5/45° samples.

The measurement of the contact angle (CA), important in the interaction of the potential medical device with the tissue, was carried out on the surfaces of the PEN, the PEN 0/22.5/45° and the Au/Ag PEN 0/22.5/45°. It is obvious from Figure 7 that the polymer with a bimetallic nanostructure exhibited a hydrophobic character. The results show an increasing contact angle with the increasing incidence angle of the laser beam during polymer irradiation. Surprisingly, the CA of laser-modified PEN (22.5° and 45°) was similar to that of pristine PEN (74.1 ± 0.3)°. The CA of the deposited samples was similar for each incidence angle, indicating that wettability could be influenced by the presence of metals. However, it cannot be excluded that nanostructured surface morphology may not play a role in the wettability of prepared samples. Because of periodical arrays, the water droplets could not be in contact with the sample surfaces absolutely as in the case of the Wenzel state. When air is trapped in gaps between nanostructured forms, the phenomenon is known as the Cassie–Baxter wetting state [40]. For this reason, a high CA was obtained, and the material appeared to be hydrophobic. The transition from the Cassie–Baxter state (meta-stable) to the Wenzel state (stable) is influenced by thermodynamic variables (temperature, pressure) and also by the parameters of the structures present on the sample surface (height and periodicity).

### 3.2. Release of Ag^+^ and Au^n+^

**Figure 8:**
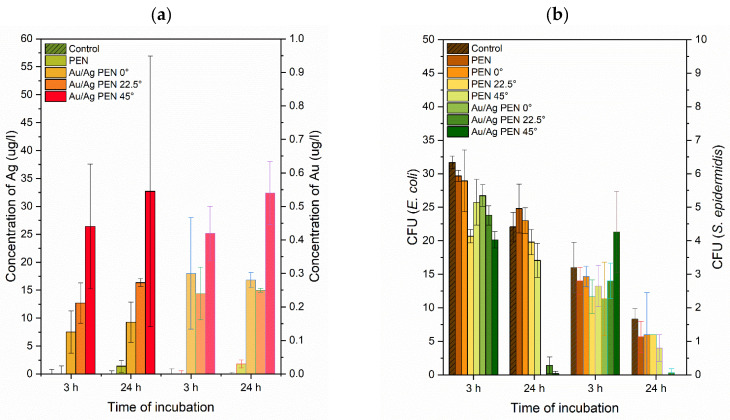
Concentrations of released Au^n+^ and Ag^+^ ions determined by ICP-MS (**a**) and data from antibacterial tests (*E. coli, S. epidermidis*) performed on PEN, PEN 0/22.5/45° and Au/Ag PEN 0/22.5/45° samples compared to control, expressed in CFU values (**b**).

Regarding the leachates of the PEN and the Au/Ag PEN 0/22.5/45° samples, concentrations of released Ag^+^ and Au^n+^ ions were determined using ICP-MS at the 3 and 24 h incubation times (Figure 8a). The physiological solution without samples and the leachate of the PEN were used as controls. The measured concentrations of metal ions increased with the increasing incidence angle of the laser beam, which probably relates to the increase of free space between the individual wires enabling better ion release in a water environment. Although the layer thicknesses of gold and silver were similar, the concentrations of Ag^+^ were much higher compared to Au^n+^. The explanation of this phenomenon is quite simple: (i) Gold is a much more noble metal compared to silver and (ii) due to characteristic metal distribution, gold was partially covered by silver on the tops of the ridges.

### 3.3. Antibacterial Tests

Antibacterial tests were conducted on PEN, PEN 0/22.5/45° and Au/Ag PEN 0/22.5/45°. Gram-negative (*Escherichia coli*) and Gram-positive (*Staphylococcus epidermidis*) bacteria were used, as they are two of the most common pathogens that participate in biomaterial-related bacterial infections. The antibacterial activity of the prepared nanowires was affected primarily by the presence of silver, which was implied from the ICP-MS analysis (Figure 8a). At an incubation time of 3 h (see Figure 8b), no antibacterial effect was observed against either bacteria strain. In this case, the effect was quite insignificant for *E. coli* and fully absent for *S. epidermidis*. While the CFU values for PEN and PEN 0/22.5/45° compared to control samples were similar, the values of the CFU for Au/Ag PEN 0/22.5/45° were slightly lower. Here a bacteria growth slowdown was observed, but not total inhibition. At the 24 h incubation time, pronounced inhibition of both bacteria was observed. The minimum inhibitory concentration (MIC) of silver ions for *E. coli* is 0.5–1 mg∙L^–1^ (dependent on the specific bacterial strain), the MIC of silver nanoparticles is 0.5–50 mg∙L^–1^ for *E. coli* and the MIC_90_ of silver nanoparticles is 6.25 mg∙L^−1^ for *S. epidermidis* (dependent on nanoparticle size) [41]. Thus, from the concentrations of released Ag^+^ ions (ICP-MS) and MIC available from the literature [41], one can see that the antibacterial effect was largely caused by the surface morphology, the roughness, and the wettability. Moreover, the CFU values for *S. epidermidis* at the 24 h incubation time were significantly lower for control and modified samples compared to the CFUs at 3 h. This phenomenon can be observed on samples of modified PEN with silver nanowires [16] and as a sputtered and annealed silver nanolayer on PI substrate [42]. *S. epidermidis* is usually more sensitive to this type of structure (with a high surface roughness) [43]. The cell walls of Gram-positive bacteria are also more permeable to positively charged particles (released metal ions) due to their composition, which is different from the cell walls of Gram-negative bacteria (*E. coli*). Therefore, the roughness considerably influenced the attachment of bacteria to the material surface.

### 3.4. Cytotoxicity

**Figure 9:**
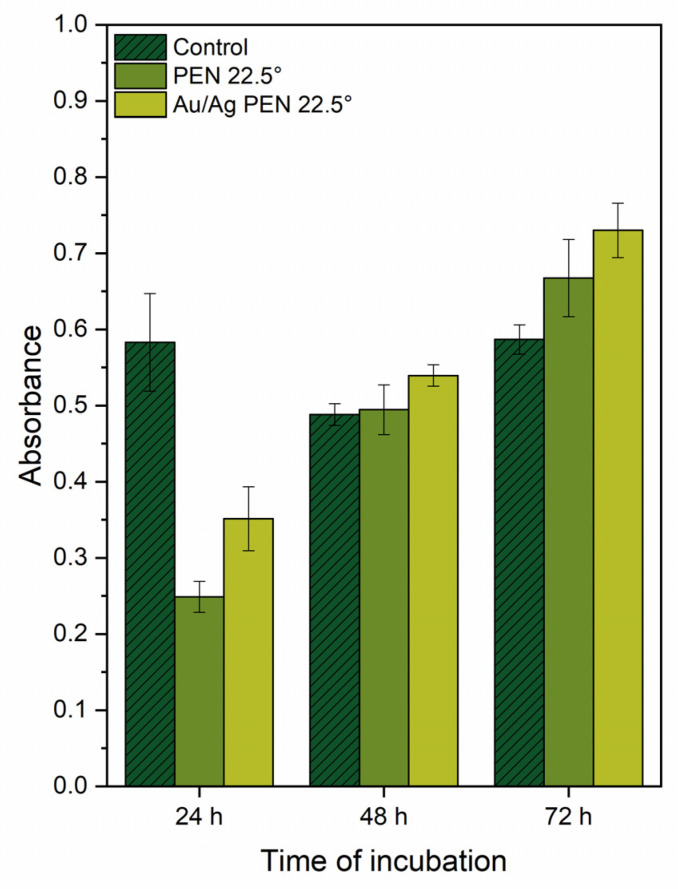
Decreasing cytotoxic effects of PEN 22.5° and Au/Ag PEN 22.5° samples, expressed by increasing absorbance.

A cytotoxic effect on human primary lung fibroblasts (Figure 9) was manifested at an incubation time of 24 h in the case of the modified samples and the modified samples with Au/AgNWs. The highest absorbance values were for control samples, which demonstrated that TCPS is an eligible material for the cultivation of eukaryotic cells [44]. The data also showed an increasing trend toward absorbance as incubation time increased for PEN 22.5° and Au/Ag PEN 22.5°, while the absorbance values after longer incubation times (48 and 72 h) were already comparable to the control and were almost two times higher than those at 24 h. The explanation for this trend may be the initially difficult adaptation of the cell line to the modified rough surface, which is important for adhesion and subsequent proliferation [45]. The fundamental influence of the polymer surface itself on the cells is evident here, as the surfaces of the modified samples were not covered with metals, and the samples showed the same trends as the samples with deposited Au/Ag nanowires. After longer incubation times (48 and 72 h), the cells apparently adapted to the rough substrate, and the cytotoxic effects disappeared.

Gold plays a key role in bimetallic nanowire behaviour during interactions with eukaryotic cells. Whereas gold cannot be considered an effective antibacterial agent, its presence is important in tissue interactions to provide biocompatibility with the final Au/AgNWs supported on the PEN. There are many studies about the toxicity of silver nanoobjects [28,46] including nanowires [27]. AgNWs produce antibacterial effects in the same bacteria strains as used in this work; unfortunately, they were found to be toxic in healthy human cells. However, the combination of silver and biocompatible metals appears to be a promising solution for decreasing the cytotoxic effects of silver. Gold deposited under the silver serves as a reducing agent because cells touch gold and silver simultaneously. In this case, the cells’ low affinity was overcome, and even cell proliferation occurred after the long incubation, which was inspired by a study on GNP polymer fibre nanocomposites [47]. Thus, preparation of a Au/Ag nanocomposite on a modified polymer surface is supposed to create an antibacterial material with a considerably low cytotoxic effect.

## 4. Conclusions

We have presented a straightforward and effective method for the preparation of bimetallic nanowires on laser-rippled PEN using a physical vapour deposition technique. The combination of laser treatment and vacuum evaporation is able to produce Au/AgNWs that are homogeneously distributed over the ripples of the modified PEN surface. AFM and SEM images confirmed the periodic nanostructures, with the surface roughness of the deposited nanowires increasing as the incidence angle increased. EDS and AR-XPS spectroscopies revealed the lamination of silver and gold on the ripple tops; the concentration of silver was significantly higher than that of gold, and it had a considerable effect on the resulting antibacterial and cytotoxic properties of the materials. Generally, the Au/AgNWs inhibited the growth of G^+^ and G^−^ bacteria and were non-toxic to healthy eukaryotic cells. Thus, the combination of these metals enabled us to develop a very promising biocompatible material with antibacterial properties that makes it highly suitable for use in medical and pharmaceutical applications.

## Figures and Tables

**Table 1:** Measured values of surface roughness (*R_a_*), periodicity (*Λ*) and height (*h*) of PEN (0/22.5/45°) and Au/Ag PEN (0/22.5/45°) nanopatterned surfaces.

Sample	*R*a (nm)	*Λ* (nm)	*h* (nm)
PEN	3.5	-	-
PEN 0°	6.7	212.4	24.8
PEN 22.5°	17.1	307.0	60.5
PEN 45°	26.3	398.0	94.8
Au/Ag PEN 0°	5.3	209.6	24.5
Au/Ag PEN 22.5°	16.2	303.4	59.4
Au/Ag PEN 45°	25.8	393.2	92.6

## Data Availability

The data presented in this study are available on request from the corresponding author.
